# Induction immunochemotherapy followed by definitive chemoradiotherapy for unresectable locally advanced non‐small cell lung cancer: a multi‐institutional retrospective cohort study

**DOI:** 10.1002/mco2.501

**Published:** 2024-03-02

**Authors:** Leilei Wu, Bo Cheng, Xiaojiang Sun, Zhenshan Zhang, Jingjing Kang, Yun Chen, Qinghua Xu, Shuangyan Yang, Yujie Yan, Shengxiang Ren, Caicun Zhou, Yaping Xu

**Affiliations:** ^1^ Department of Radiation Oncology Shanghai Pulmonary Hospital School of Medicine Tongji University Shanghai China; ^2^ Department of Radiation Oncology Cancer Hospital of University of Chinese Academy of Sciences (Zhejiang Cancer Hospital) Institute of Cancer and Basic Medicine (IBMC) Chinese Academy of Sciences Hangzhou China; ^3^ Department of Radiation Oncology Qilu Hospital Cheeloo College of Medicine Shandong University Jinan China; ^4^ Department of Radiation Oncology Shanghai Proton and Heavy Ion Center Fudan University Cancer Hospital Shanghai China; ^5^ Department of Medical Oncology Shanghai Pulmonary Hospital School of Medicine Tongji University Shanghai China

**Keywords:** definitive chemoradiotherapy, Induction immunochemotherapy, survival, unresectable LA‐NSCLC

## Abstract

This study aimed to evaluate the efficacy and safety of induction immunochemotherapy followed by definitive chemoradiotherapy (CRT) for unresectable locally advanced non‐small cell lung cancer (LA‐NSCLC). We identified unresectable stage III NSCLC patients who received induction immunochemotherapy. Overall survival (OS) and progression‐free survival (PFS) were the primary endpoints. From February 2019 to August 2022, 158 patients were enrolled. Following the completion of induction immunochemotherapy, the objective response rate (ORR) and disease control rate (DCR) were 52.5% and 83.5%, respectively. The ORR of CRT was 73.5%, representing 68.4% of the total cohort. The median PFS was 17.8 months, and the median OS was 41.9 months, significantly higher than in patients who received CRT alone (*p* < 0.001). Patients with concurrent CRT demonstrated markedly improved PFS (*p* = 0.012) and OS (*p* = 0.017) than those undergoing sequential CRT. Additionally, those with a programmed‐death ligand 1 (PD‐L1) expression of 50% or higher showed significantly elevated ORRs (72.2% vs. 47.2%, *p* = 0.011) and superior OS (median 44.8 vs. 28.6 months, *p* = 0.004) compared to patients with PD‐L1 expression below 50%. Hematologic toxicities were the primary severe adverse events (grade ≥ 3) encountered, with no unforeseen treatment‐related toxicities. Thus, induction immunochemotherapy followed by definitive CRT demonstrated encouraging efficacy and tolerable toxicities for unresectable LA‐NSCLC.

## Graphical abstract

We assessed the efficacy and safety of induction immunochemotherapy followed by definitive chemoradiotherapy (CRT) for unresectable stage III locally advanced non‐small cell lung cancer. Of the 158 patients enrolled, compared to CRT alone, results showed significantly improved overall survival and progression‐free survival, particularly in patients with programmed‐death ligand 1 expression of 50% or higher. No unexpected treatment‐related toxicities were observed. The treatment demonstrated encouraging efficacy and tolerable toxicities.

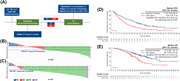

## 1 INTRODUCTION

Lung cancer continues to be the foremost cause of death related to cancer globally, accounting for approximately 1.8 million fatalities each year.^1^ Non‐small cell lung cancer (NSCLC) constitutes approximately 85% of all lung cancer cases, with approximately one‐third of NSCLC patients presenting with locally advanced (LA) stage at diagnosis.^2^ Consolidation with immune checkpoint inhibitors (ICIs) has shifted the treatment paradigm for unresectable LA‐NSCLC away from concurrent chemoradiotherapy (CRT).^3^, ^4^, ^5^ Updated results from the PACIFIC trial demonstrated that the addition of the consolidation ICI after concurrent CRT significantly prolonged overall survival (OS) to 47.5 months, compared with 29.1 months in the placebo group.^6^ The GEMSTONE‐301 trial further supported the benefit of ICI consolidation, indicating a marked improvement in progression‐free survival (PFS) after either concurrent or sequential CRT.^7^ Unfortunately, despite these advances, nearly half of the patients with unresectable LA‐NSCLC are unable to receive consolidation ICI after concurrent CRT, which is associated with a dismal prognosis.^8^

Logically, adjusting ICI from the consolidation phase to the induction setting could significantly enhance patient adherence to ICI treatment. Moreover, induction treatment with ICI has the potential to diminish tumor size, which could further reduce the target volume for subsequent radiotherapy and result in lower toxicity.^9^ Contrasting with the PACIFIC trial, the GEMSTONE‐301 trial, which included more stage IIIB and IIIC patients, demonstrated inferior survival outcomes, even inferior to concurrent CRT alone, suggesting that consolidation ICI may not yield the efficacy for bulky LA‐NSCLC as indicated in the PACIFIC trial.^6^, ^10^, ^11^ Therefore, radiation oncologists facing the challenge of larger tumor sizes might favor induction immunochemotherapy to diminish the tumor mass, facilitating a safer and more effective administration of definitive CRT. Such an induction strategy might provide an equal or possibly improved therapeutic benefit relative to consolidation therapy.

Additionally, induction immunochemotherapy plays a role in achieving pathological downstaging and improving survival before surgery.^12^ There is also a debate about whether induction immunochemotherapy can convert unresectable stage III NSCLC to resectable. Consequently, an increasing number of patients are entering this treatment pathway, but many may ultimately miss the opportunity for radical surgery.^13^, ^14^ The vast majority would inevitably receive definitive CRT, thus from this perspective, the outcomes of induction ICI with chemotherapy followed by CRT warrant further investigation.

Here, we performed a retrospective study to evaluate the efficacy and safety of induction immunochemotherapy followed by definitive CRT for unresectable stage III LA‐NSCLC. We enrolled patients who were ineligible for radical surgery after induction immunochemotherapy, those undergoing induction immunochemotherapy with the intent of downsizing initial tumors to enable subsequent definitive CRT, as well as those from prospective clinical trials investigating induction immunochemotherapy followed by definitive CRT.

## 2 RESULTS

### 2.1 Patient characteristics

**FIGURE 1:**
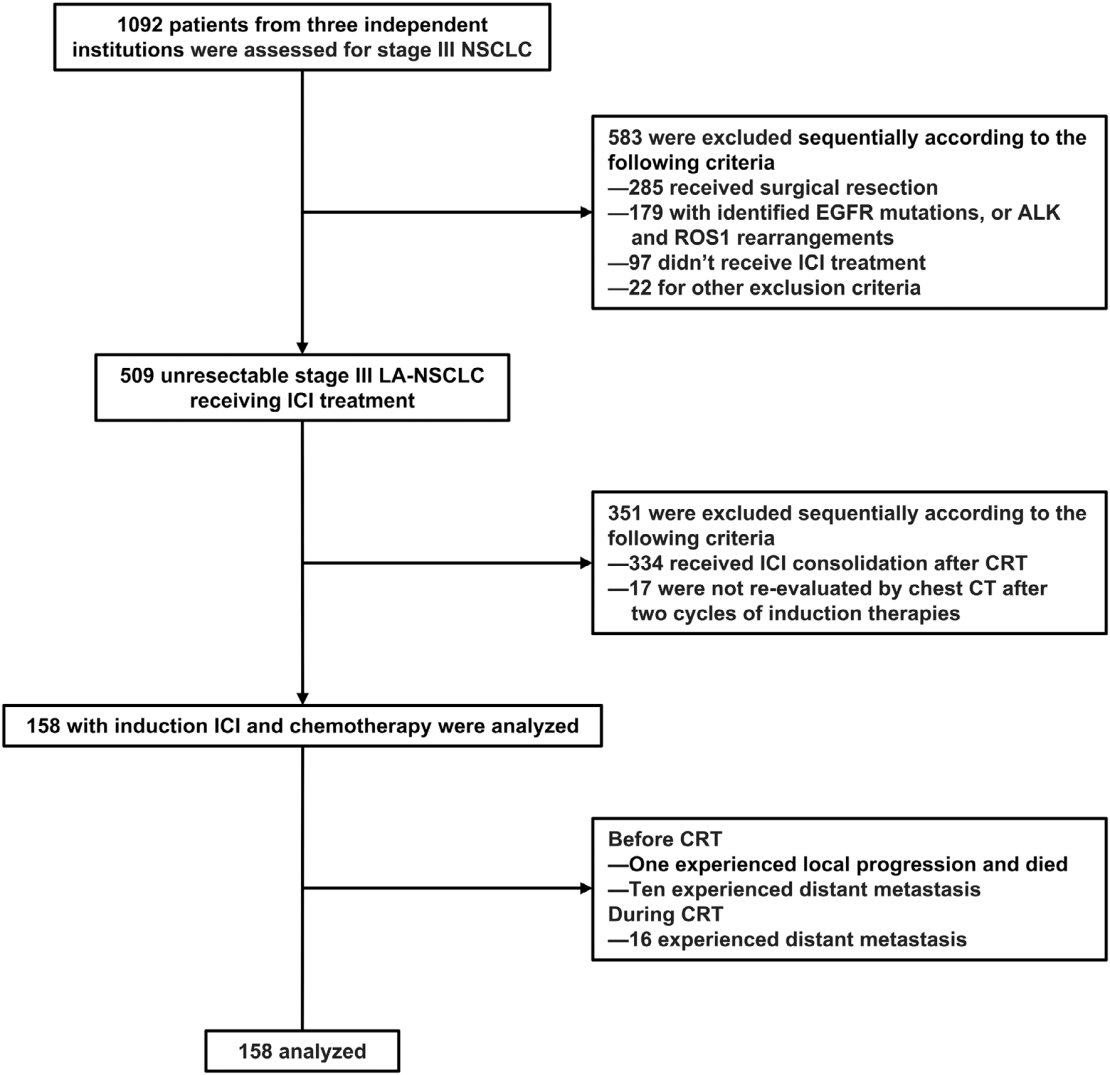
Flowchart of eligible patient selection. CRT, chemoradiotherapy; ICI, immune checkpoint inhibitors; LA, locally advanced; NSCLC, non‐small cell lung cancer.

From February 2019 to August 2022, 1,092 stage III LA‐NSCLC patients from three institutions were assessed. Of these, 583 patients (53.4%) were excluded following this sequence of criteria: 285 for resectable diseases, 179 with *EGFR* mutations, or *ALK* and *ROS1* rearrangements, 97 who didn't receive ICI treatment, and 22 for other exclusion criteria. In total, 509 patients with stage III LA‐NSCLC received ICI treatment; 334 of whom received ICI consolidation after CRT, and 17 were not re‐evaluated by chest CT after two cycles of induction therapies. Ultimately, the final analysis included 158 patients who received induction ICI combined with chemotherapy (refer to Figure 1 for details). The baseline characteristics of these patients are detailed in Table 1. It is noteworthy that the baseline characteristics of the patients who received only CRT, used for comparison of survival and toxicity with the target population, are also summarized in Table 1. Significant differences were not observed in the baseline characteristics across the two patient groups. Of all patients, 89 (56.3%) were under 65 years of age, 143 (90.5%) were male, 119 (75.3%) had a smoking history, and 55 (34.8%) had an Eastern Cooperative Oncology Group (ECOG) performance status of 0. The histological subtypes included squamous cell carcinoma in 95 (60.1%), non‐squamous cell carcinoma in 51 (32.3%), and not otherwise specified in 12 (7.6%) patients. The stages were distributed as follows: 49 (31.0%) with stage IIIA, 67 (42.4%) with stage IIIB, and 42 (26.6%) with stage IIIC. Programmed death ligand‐1 (PD‐L1) expression of 50% or more was found in 36 (22.8%) patients and PD‐L1 expression between 1% and 50% in 42 (26.6%) patients; 47 (29.7%) patients had PD‐L1 expression less than 1%, and the PD‐L1 status was unknown for 33 (20.9%) patients. Notably, five *EGFR*‐mutant and two *ALK*‐rearranged patients were discovered after disease progression.

**TABLE 1:** Baseline characteristics of 158 enrolled patients and 97 patients who received only CRT.

	Patients (*n* = 255)		
Characteristic	Induction (*n* = 158)	CRT alone (*n* = 97)	*p*‐Value
Age, years			
Median	63 (56‐68)		
< 65	89 (56.3)	59 (60.8)	0.480
≥65	69 (43.7)	38 (39.2)	
Sex			
Male	143 (90.5)	82 (84.5)	0.151
Female	15 (9.5)	15 (15.5)	
Smoking history			
Never smoked	39 (24.7)	30 (30.9)	0.276
Former or current smoker	119 (75.3)	67 (69.1)	
ECOG performance status			
0	55 (34.8)	25 (25.8)	0.176
1	94 (59.5)	62 (63.9)	
2	9 (5.7)	10 (10.3)	
Tumor histological type			
Squamous cell carcinoma	95 (60.1)	53 (54.6)	0.140
Non‐squamous cell carcinoma	51 (32.3)	29 (29.9)	
Missing data	12 (7.6)	15 (15.5)	
Disease stage			
IIIA	49 (31.0)	39 (40.2)	0.208
IIIB	67 (42.4)	40 (41.2)	
IIIC	42 (26.6)	18 (18.6)	
PD‐L1 expression^†^			
< 1%	47 (29.7)	35 (36.1)	0.078
1‐49%	42 (26.6)	23 (23.7)	
≥50%	36 (22.8)	11 (11.3)	
Missing	33 (20.9)	28 (28.9)	

Data are median (IQR) or *n* (%).

Abbreviations: CRT, chemoradiotherapy; ECOG, Eastern Cooperative Oncology Group; IQR, interquartile ranges; PD‐L1, programmed death ligand‐1.

† Assessment of baseline PD‐L1 expression was not mandatory for clinical treatment or enrollment in the study, consequently leading to the absence of PD‐L1 status in over 20% of patients who were included.

### 2.2 Treatment outcomes

Treatment‐related information for all patients is detailed in Table 2. The induction immunochemotherapy cycles ranged from two to eight, with the vast majority (88.0%) of patients undergoing up to four cycles. Specifically, 27 (17.1%) patients received two cycles, 68 (43.0%) received three cycles, and 44 (27.8%) received four cycles of induction immunochemotherapy. The decision to extend therapy beyond four cycles, either for an additional cycle (4.4%) or two cycles (6.3%), was made based on the treatment response and patient preferences, as determined by the overseeing oncologists. There were instances where two patients received extended cycles of induction immunochemotherapy due to unavoidable delays in curative radiotherapy and strong patient requests; one patient received seven cycles and another eight cycles, with a switch to monotherapy with ICI after four cycles of immunochemotherapy. In the group of 147 patients who completed both induction immunochemotherapy and CRT, a higher proportion received radiation doses above 60 Gy (62.6%) and underwent sequential CRT (63.3%). Among those without distant metastasis after CRT (*n* = 131), 20 did not proceed to further consolidation treatment, while 62 (47.3%) completed their consolidation treatment within 1 year.

**TABLE 2:** Summary of treatment and response to treatment.

	Total (*n* = 158)
Cycles of induction ICI	*n* = 158
2	27 (17.1)
3	68 (43.0)
4	44 (27.8)
5	7 (4.4)
6	10 (6.3)
7	1 (0.6)
8	1 (0.6)
Radiotherapy	*n* = 147^†^
< 60 Gy	55 (37.4)
≥60 Gy	92 (62.6)
Sequence of CRT	*n* = 147
Sequential	93 (63.3)
Concurrent	54 (36.7)
Duration of consolidation ICI	*n* = 131^‡^
None	20 (15.3)
≤12 months	62 (47.3)
13–18 months	40 (30.5)
19–24 months	9 (6.9)
The best response to induction immunochemotherapy	*n* = 158
CR	1 (0.6)
PR	82 (51.9)
SD	49 (31.0)
PD	26 (16.5)
Best response to CRT	*n* = 147
CR	3 (2.0)
PR	105 (71.4)
SD	16 (10.9)
PD	23 (15.7)

Data are *n* (%).

Abbreviations: CR, complete response; CRT, chemoradiotherapy; ICI, immune checkpoint inhibitors; PD, progressive disease; PR, partial response; SD, stable disease.

† Ten patients who developed distant metastases and one patient who experienced local progression and death during induction therapy did not meet the criteria for definitive radiotherapy and therefore were excluded from this segment.

‡ Patients who died or progressed before the completion of CRT did not meet the criteria for consolidation treatment and were therefore excluded from this segment.

**FIGURE 2:**
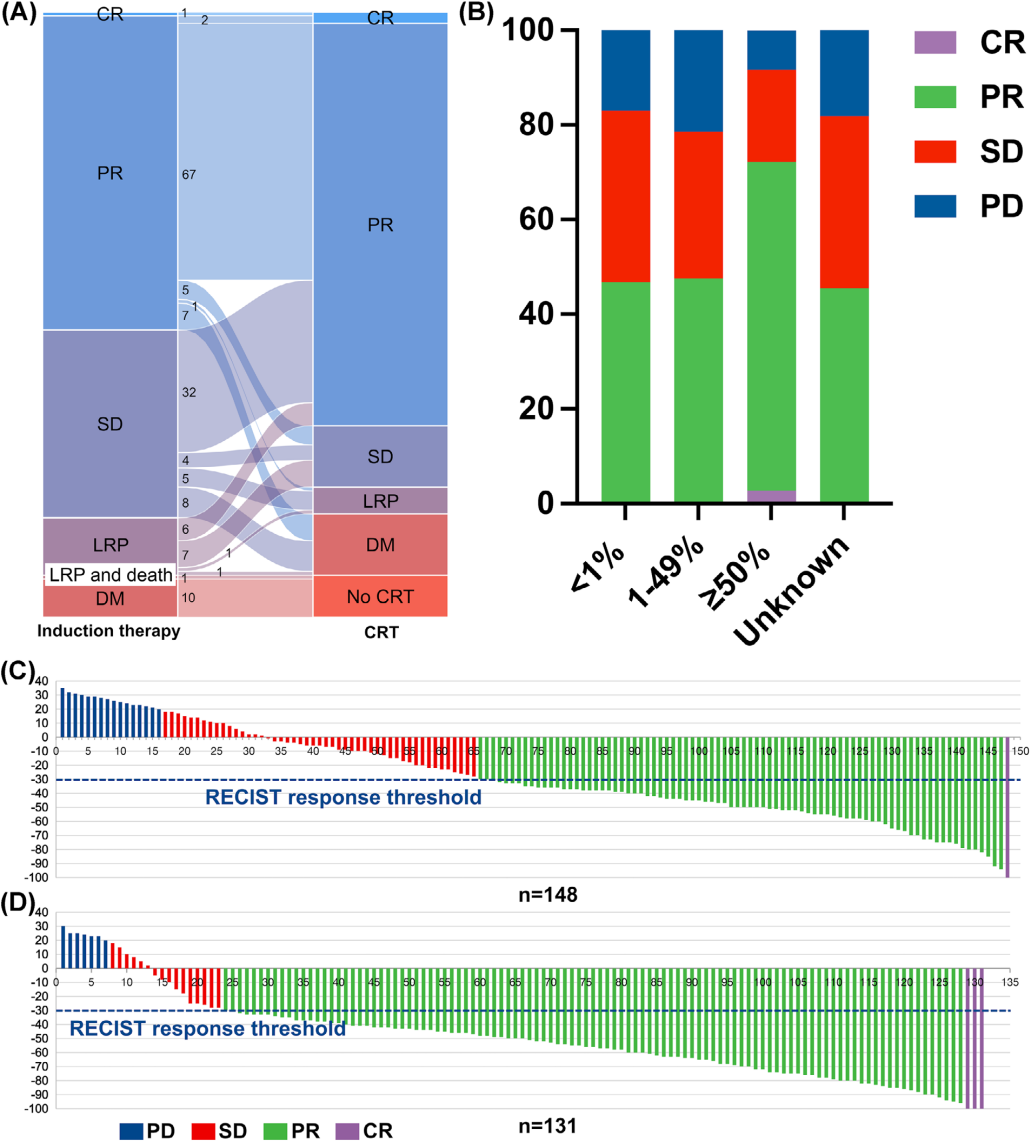
Best response to induction immunochemotherapy and CRT. (A) Sankey plot depicting treatment response changes for each patient from induction immunochemotherapy to CRT. (B) The best response to induction immunochemotherapy based on different baseline PD‐L1 expression statuses. (C) Waterfall chart depicting best tumor responses for all 148 patients with induction immunochemotherapy with no metastasis. (D) Waterfall chart depicting best tumor responses for all 131 patients treated with CRT with no metastasis. CR, complete response; CRT, chemoradiotherapy; DM, distant metastasis; LRP, local‐regional progression; PD, progressive disease; PD‐L1, programmed death ligand‐1; PR, partial response; SD, stable disease.

In the induction immunochemotherapy cohort, one patient achieved complete response (CR) and 82 patients reached partial response (PR). Progression was observed in 26 patients, including 16 with local‐regional progression and 10 with distant metastasis. Notably, among the patients with progression, one experienced local‐regional advancement and eventually succumbed to pneumonitis related to ICI. Consequently, 147 patients proceeded to curative intent CRT. Post‐CRT evaluations revealed that three individuals achieved CR, and 105 achieved PR, resulting in an objective response rate (ORR) of 73.5%. This ORR represents 68.4% of the initial cohort of 158 patients at baseline. Regrettably, there were still 23 patients who showed progression compared to baseline, with seven exhibiting local‐regional progression and 16 distant metastasis. The detailed treatment response categories for all patients throughout the immunochemotherapy are presented in Figure 2. Figure 2A depicts the treatment response changes for each patient from induction immunochemotherapy to definitive CRT. As for the comparative response to induction immunochemotherapy, these were analyzed based on the baseline tumor PD‐L1 expression status, using cutoffs of 1% and 50% (Figure 2B). The ORR was significantly higher in patients with PD‐L1 expression above 50%, compared to those with less than 50% (72.2% vs. 47.2%, *p* = 0.011). However, this difference was not statistically significant when a 1% cutoff was used (46.8% vs. 59.0%, *p* = 0.186; Table S1). The best responses to induction immunochemotherapy (*n* = 158) and CRT (*n* = 131), excluding those with distant metastasis, are detailed in Figure 2C,D, with all assessments made relative to the baseline.

### 2.3 Survival

**FIGURE 3:**
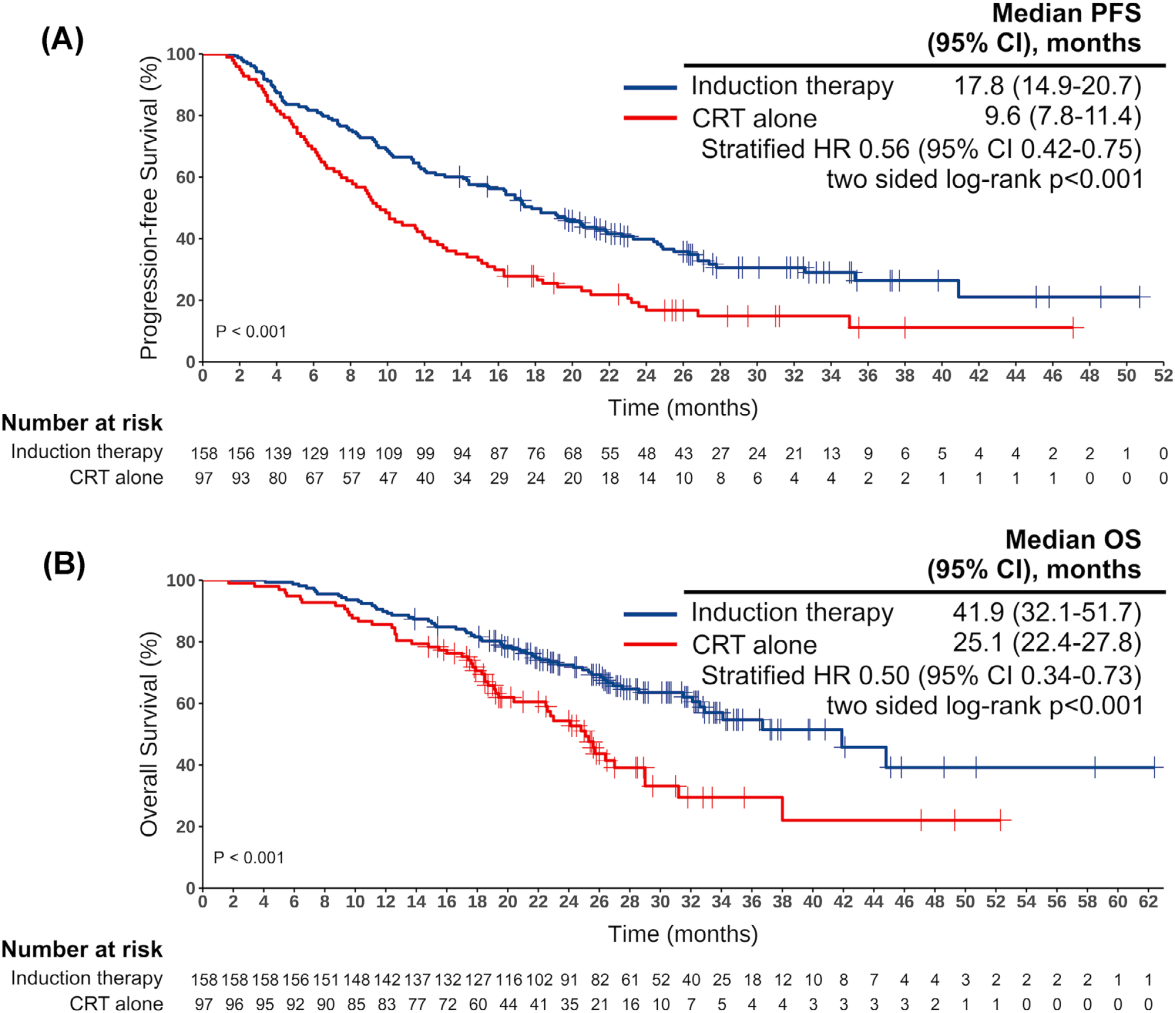
Comparison of survival curves between the target cohort receiving induction immunochemotherapy and the cohort receiving CRT alone. (A) Comparison of PFS between the two groups. (B) Comparison of OS between the two groups. CI, confidence interval; HR, hazard ratio; OS, overall survival; PFS, progression‐free survival.

**FIGURE 4:**
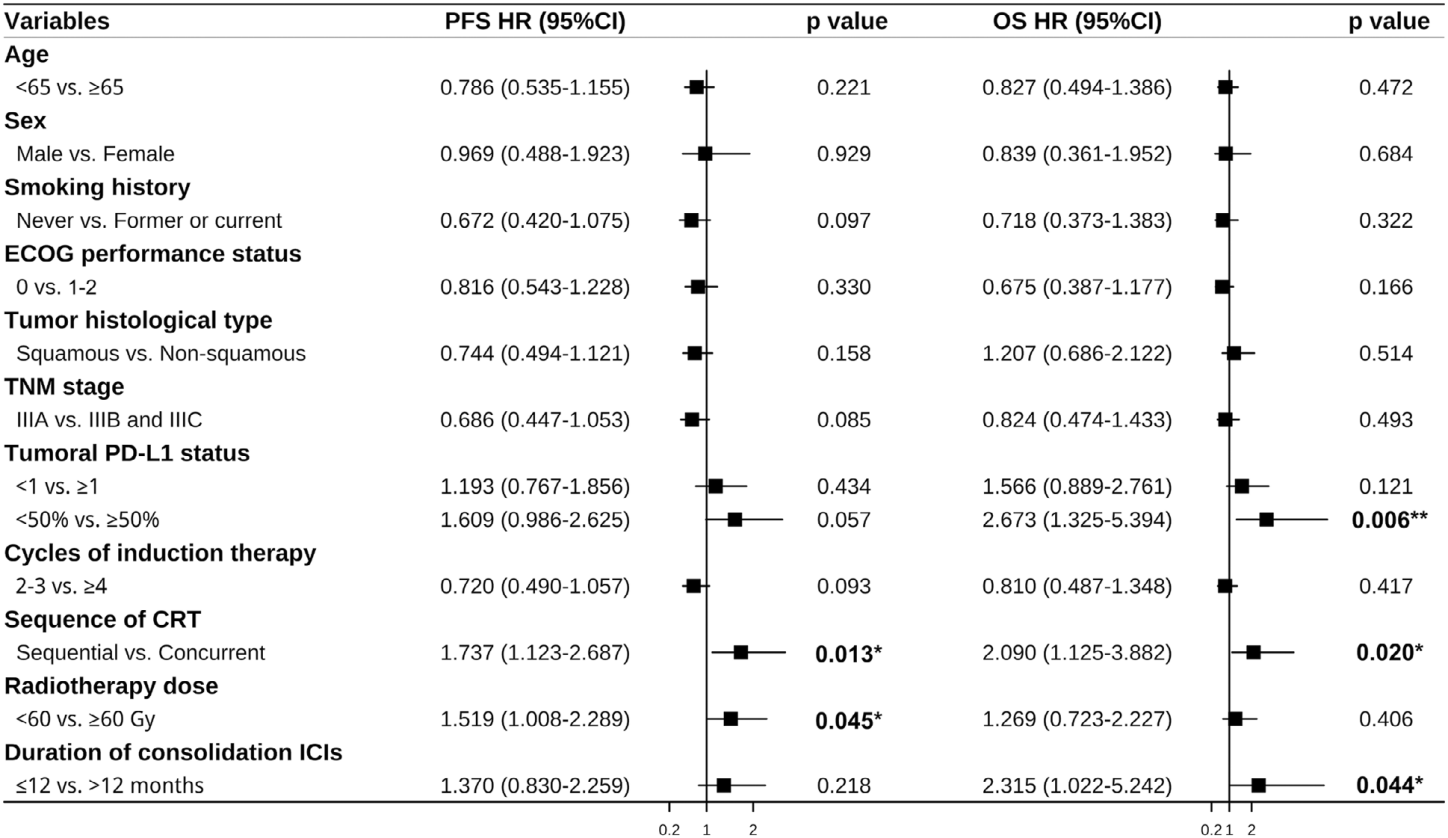
Univariate analysis of PFS and OS. CI, confidence interval; CRT, chemoradiotherapy; ECOG, Eastern Cooperative Oncology Group; HR, hazard ratio; ICI, immune checkpoint inhibitors; OS, overall survival; PD‐L1, programmed death ligand‐1; PFS, progression‐free survival. *p < 0.05, **p < 0.01.

Considering the cohort of patients who underwent only CRT, the median follow‐up period for the entire study population was 28.9 months (interquartile ranges [IQR] 23.4–33.9). As of October 15, 2023, 52 and 16 patients in the two respective study groups had not yet experienced any progression of their disease. Within our target population, a total of 49 patients who completed ICI consolidation treatment had not encountered any disease progression, and three patients were still undergoing consolidation treatment. The introduction of induction immunochemotherapy markedly improved PFS compared to patients who received CRT alone (Hazard ratio [HR] 0.56, 95% confidence intervals [CI] 0.42–0.75; two‐sided *p* < 0.001; Figure 3A). The median PFS was 17.8 months (95% CI 14.9–20.7) in the induction immunochemotherapy group versus 9.6 months (95% CI 7.8–11.4) in the CRT‐alone group. In the induction immunochemotherapy cohort, PFS rates at 1, 3, and 4 years were 62.0%, 26.4%, and 21.1%, respectively, compared with 40.2% and 11.1% at 1 and 3 years in the CRT‐alone group. OS was also significantly prolonged in patients receiving induction immunochemotherapy compared to those who underwent only CRT, with a median OS of 41.9 months (95% CI 32.1–51.7) versus 25.1 months (95% CI 22.4–27.8) (HR 0.50, 95% CI 0.34‐0.73; two‐sided p < 0.001; Figure 3B). For the induction immunochemotherapy group, the 1‐, 3‐, and 5‐year OS rates were 89.9%, 54.7%, and 39.2%, respectively. In contrast, the CRT‐alone group exhibited 1‐ and 3‐year survival rates of 85.6% and 29.4%, respectively, with the 5‐year survival rate not yet reached within the follow‐up period. The survival curve comparisons for some subgroups are presented in Figure S1. Notably, for those patients who successfully underwent CRT following induction immunochemotherapy, an impressive 5‐year OS rate of 41.9% was observed (Figure S1B). Additionally, the median PFS and OS were 19.6 and 41.9 months, respectively (Figure S1A,B). Similar to the treatment responses, patients with PD‐L1 expression over 50% had significantly longer OS compared to those with less than 50% (median 44.8 months vs. 28.6 months; two‐sided *p* = 0.004; Figure S1D). A near‐significant difference was also observed in PFS (*p* = 0.055; Figure S1C). However, these differences were not statistically significant when a PD‐L1 expression cutoff of 1% was used. Patients receiving concurrent CRT manifested significantly better PFS (*p* = 0.012; Figure S1E) and OS (*p* = 0.017; Figure S1F), compared to those treated with sequential CRT. PFS and OS were further analyzed and compared across various variables through univariate analysis (Figure 4). Variables with a *p*‐value < 0.1 were included in the multivariate analysis, and missing values were excluded. Six variables were incorporated into the multivariate analysis for PFS, covering 116 cases, while three factors were included for OS analysis, encompassing 87 cases. The multivariate analysis revealed that non‐smoking status, 2–3 cycles of induction immunochemotherapy, and concurrent CRT were significant prognostic factors for longer PFS. Only concurrent CRT emerged as a significant prognostic factor for extended OS (Table S2).

### 2.4 Safety

The comparison of treatment‐related toxicities between patients who underwent induction ICI combined with chemotherapy followed by CRT and those who received CRT alone is presented in Table 3. In the induction group, the incidence rates of grade 1–2, 3–4, and 5 pneumonitis or radiation pneumonitis (RP) were 31.0%, 4.4%, and 1.3%, respectively, compared to 26.8%, 3.1%, and 1.0% in the CRT‐alone group, showing no significant differences. One patient in the induction group died from immune‐related pneumonitis during induction treatment, and another died from RP after completing CRT, while one patient in the CRT‐alone group died from RP. All patients experiencing grade 2 or grade 3 RP had fully recovered after treatment with oral or intravenous corticosteroids. Generally, most of the treatment‐related toxicities in both groups were mild, predominantly with grades 1–2. Induction immunochemotherapy only led to a near‐significant increase in hypothyroidism and hyperthyroidism, which are relatively easy to manage clinically. Other toxicities showed no significant differences between the two groups. Among patients who received induction immunochemotherapy, radiation esophagitis was the most common in grade 1–2 toxicities (44.2%), followed by leukopenia (43.7%) and fatigue (39.9%). The most frequent severe (grade ≥3) adverse events were hematologic toxicities, including leukopenia (8.9%) and thrombocytopenia (6.3%).

**TABLE 3:** Comparison of treatment‐related toxicities between induction therapy and chemoradiotherapy (CRT) alone.

	Grade 1–2			Grade 3–4			Grade 5		
Toxicity	Induction (*n* = 158)	CRT alone (*n* = 97)	p‐Value	Induction (*n* = 158)	CRT alone (*n* = 97)	p‐Value	Induction (*n* = 158)	CRT alone (*n* = 97)	p‐Value
Any event^†^	108 (68.4)	59 (60.8)	0.220	31 (19.6)	15 (15.5)	0.402	2 (1.3)	1 (1.0)	1.000
Pneumonitis or RP^‡^	49 (31.0)	26 (26.8)	0.474	7 (4.4)	3 (3.1)	0.746	2 (1.3)	1 (1.0)	1.000
Radiation esophagitis^§^	65 (44.2)	37 (38.1)	0.636	1 (0.6)	1 (1.0)	1.000	0 (0.0)	0 (0.0)	1.000
Hypothyroidism	26 (16.5)	8 (8.2)	0.061	1 (0.6)	0 (0.0)	1.000	0 (0.0)	0 (0.0)	1.000
Hyperthyroidism	21 (13.3)	5 (5.2)	0.053	1 (0.6)	1 (1.0)	1.000	0 (0.0)	0 (0.0)	1.000
Gastrointestinal reaction	58 (36.7)	30 (30.9)	0.346	2 (1.3)	0 (0.0)	0.527	0 (0.0)	0 (0.0)	1.000
Fatigue	63 (39.9)	29 (29.9)	0.107	0 (0.0)	0 (0.0)	1.000	0 (0.0)	0 (0.0)	1.000
Anemia	8 (5.1)	4 (4.1)	1.000	4 (2.5)	3 (3.1)	1.000	0 (0.0)	0 (0.0)	1.000
Leukopenia	69 (43.7)	36 (37.1)	0.302	14 (8.9)	6 (6.2)	0.441	0 (0.0)	0 (0.0)	1.000
Thrombocytopenia	52 (32.9)	27 (27.8)	0.407	10 (6.3)	5 (5.2)	0.790	0 (0.0)	0 (0.0)	1.000
Lymphopenia	13 (8.2)	6 (6.2)	0.547	6 (3.8)	3 (3.1)	1.000	0 (0.0)	0 (0.0)	1.000
Transaminase elevation	15 (9.5)	7 (7.2)	0.530	4 (2.5)	2 (2.1)	1.000	0 (0.0)	0 (0.0)	1.000
Serum amylase elevation	5 (3.2)	3 (3.1)	0.975	3 (1.9)	1 (1.0)	1.000	0 (0.0)	0 (0.0)	1.000

Data are *n* (%).

Abbreviations: CRT, chemoradiotherapy; RP, radiation pneumonitis.

† Included are events that were reported in at least 10% of the patients in either group. Grade 5 adverse events of any cause occurred in two patients who received neoadjuvant therapy; one died of immune‐related pneumonitis during the induction phase, and another succumbed to RP following the completion of CRT. Additionally, in the group receiving only CRT, there was one case of fatality due to RP.

‡ In clinical practice, distinguishing between pneumonia and radiation pneumonitis can be challenging, therefore, these conditions were combined for comparison purposes here. This refers to the proportion among all patients who underwent neoadjuvant treatment (*n* = 158).

§ The incidence rate in the induction group refers to the proportion of patients who underwent radiotherapy (*n* = 147).

## 3 DISCUSSION

To the best of our knowledge, this multi‐institutional retrospective study is the largest to date examining the efficacy and safety of induction ICI with chemotherapy followed by definitive CRT in patients with unresectable LA‐NSCLC. The results of this innovative treatment approach are promising, evidenced by a median PFS of 17.8 months and a median OS of 41.9 months. Notably, while severe adverse events were primarily hematologic, the overall treatment was well‐tolerated, underscoring its favorable safety profile.

The therapeutic landscape for LA‐NSCLC underwent a paradigm shift with the advent of immunotherapy. Previous strategies, such as post‐CRT chemotherapy^15^ or targeted therapy consolidations,^16^ failed to yield significant survival improvements. The breakthrough of the PACIFIC trial not only set a new standard for treating unresectable LA‐NSCLC but also established a foundation for post‐CRT ICI consolidation therapy.^4^, ^6^ Echoing these findings, the GEMSTONE‐301 trial demonstrated a notable PFS extension with sugemalimab consolidation compared to placebo (10.5 vs. 6.2 months, *p* = 0.0012).^7^ While the concurrent CRT arm showed a median PFS of 15.7 months, surpassing the 8.1 months in the sequential CRT arm, this underscores the broader potential patient benefit from ICI. Nonetheless, almost half of the unresectable stage III NSCLC patients receiving CRT were ineligible for durvalumab as per PACIFIC criteria.^8^, ^17^, ^18^ Integrating ICI with concurrent CRT could extend ICI benefits to all patients fit for concurrent CRT, potentially harnessing synergies between chemotherapy and ICI.^19^, ^20^ This approach's viability and safety were supported by the phase II trial KEYNOTE‐799, paving the way for further advancements in LA‐NSCLC treatment strategies.^21^, ^22^

Induction ICI, alone or with chemotherapy followed by CRT, extends the benefits of ICI treatment to more patients. This approach potentially utilizes a more intact immune system, undiminished by CRT effects on lymphocytes and stem cells.^23^, ^24^ The AFT‐16 phase II trial explored this, starting with two cycles of atezolizumab, followed by restaging and additional treatment if no progression occurred.^25^ Results included a 77.4% DCR at 12 weeks and a median PFS of 23.7 months, with PFS rates of 66% and 57% at 12 and 18 months, respectively. The 18‐month OS rate was 84%. Compared to the 55% 12‐month PFS in the PACIFIC trial, the AFT‐16's 78% post‐concurrent CRT PFS is noteworthy. Although the population was highly selected, the trial broadened eligibility beyond concurrent CRT responders.^4^, ^6^, ^26^ Combining ICI with chemotherapy may synergistically amplify the effects of induction treatment. A recent single‐center retrospective study by Bi et al. on patients with bulky unresectable stage III NSCLC treated with induction ICI and chemotherapy followed by definitive CRT, who received ICI and chemotherapy as induction treatment followed by definitive CRT, reported an ORR of 76.1% for the induction phase and 86.7% after CRT.^11^ This outcome even surpasses the triplet regimen in the KEYNOTE‐799 study,^21^, ^22^ with a median PFS of 30.6 months. However, the study excluded patients with distant metastasis during induction immunochemotherapy and CRT, and those receiving less than 60 Gy radiation, limiting its representativeness for all patients undergoing induction immunochemotherapy. Our study, encompassing multiple centers and a larger patient cohort, included all patients receiving induction immunochemotherapy. We also compared survival and toxicity data against patients only treated with CRT, ensuring a comprehensive and unbiased analysis. Notably, results from patients of two centers who received definitive CRT after induction immunochemotherapy and had no distant metastasis before the end of CRT were previously accepted as a poster at the World Conference on Lung Cancer.^27^

Our study reports a median PFS of 17.8 months and a median OS of 41.9 months in unresectable stage III NSCLC, surpassing many similar studies without selective criteria. While cross‐trial comparisons in unresectable stage III NSCLC among different treatment modalities are inherently complex due to varying ICI timing and patient selection, our results hint at the potential superiority of this treatment approach. In subgroup analyses, the PACIFIC trial indicated limited benefit from durvalumab consolidation in patients with PD‐L1 expression below 25% and even lesser effectiveness for those with PD‐L1 under 1%.^6^ This finding is consistent with a 2022 ASCO study indicating reduced survival in stage III unresectable NSCLC patients with PD‐L1 under 1% after concurrent CRT.^28^ Our study found notably higher ORR and OS in patients with over 50% PD‐L1 expression, while those with less than 1% showed poorer outcomes, albeit not statistically significant. These findings highlight the need for PD‐L1 level‐based personalized treatments. We also observed that concurrent CRT following induction immunochemotherapy enhances survival more than sequential CRT, aligning with GEMSTONE‐301 results.^10^ Additionally, similar to the study of Bi et al.,^11^ our data suggest that 2–3 cycles of induction immunochemotherapy might be optimal, as longer induction could delay CRT and worsen prognosis, whereas prolonged consolidation therapy post‐CRT seems advantageous.

In the PACIFIC trial, patients receiving consolidation durvalumab exhibited a 33.9% incidence of all‐grade pneumonitis and 4.2% for grade 3–4 pneumonitis.^4^ A real‐world study reported 35% for all grades and 6% for grade ≥3 pneumonitis.^29^ In trials like NICOLAS^30^ and KEYNOTE‐799,^21^ synchronous ICI and CRT led to grade ≥3 pneumonitis incidences between 6.9% and 11.7%. Our study observed 31.0% of patients with grade 1–2 pneumonitis or RP, and a 5.7% incidence for grade 3 or higher. The lower incidence of severe pulmonary toxicities in the PACIFIC trial might be due to the exclusion of patients with grade 2 or higher pneumonitis from prior concurrent CRT, thus removing those more susceptible to pulmonary toxicity. Concurrent ICI with CRT in other trials may have increased severe pulmonary toxicities due to the combined treatment effects. Conversely, the objective response in over half of our cohort post‐induction immunochemotherapy led to smaller irradiation fields, contributing to lower pulmonary toxicity rates. Therefore, induction immunochemotherapy followed by definitive CRT could be more advantageous due to its lower pulmonary toxicity profile.

This study has several limitations. First, despite being the largest multi‐center study reported to date, the overall number of centers and sample size is still limited, necessitating further validation across more centers and populations. Second, to focus on the target population, patients who underwent surgery, had mutations or received consolidation immunotherapy were excluded. Thus, outcomes for the broader stage III population and comparisons of different treatment modalities require further exploration. Third, studies like PACIFIC and GEMSTONE‐301 excluded patients who progressed during CRT and started follow‐up from the commencement of consolidation treatment, making direct comparisons between these treatment modalities challenging. Fourth, PFS may be overestimated, as treatment assessments in real‐world practice can sometimes be delayed. Fifth, the retrospective nature of the study introduces inherent limitations, including selection bias and numerous confounding factors, underscoring the need for prospective studies to validate these findings. Sixth, some patients had missing baseline characteristics and treatment data, which led to their exclusion from the regression analyses, introducing bias and affecting the accuracy of the results.

## 4 CONCLUSION

In conclusion, the combination of induction immunochemotherapy and subsequent definitive CRT presents an effective and manageable treatment approach for patients with unresectable LA‐NSCLC, positioning it as a viable alternative treatment strategy for this patient population.

## 5 MATERIALS AND METHODS

### 5.1 Study design and patient population

This is a multi‐institutional retrospective cohort study, where electronic medical records of stage III NSCLC patients from February 2019 to August 2022 at Shanghai Pulmonary Hospital, Zhejiang Cancer Hospital, and Shandong University Qilu Hospital were reviewed. Patients eligible for inclusion met the following criteria: (1) histologically confirmed stage III (IIIA, IIIB, or IIIC) NSCLC and clinically unresectable LA disease according to the 8th edition of the American Joint Committee on Cancer cancer staging manual^31^, ^32^; (2) receiving two or more cycles of induction immunochemotherapy with ICI combined with chemotherapy; (3) having comprehensive electronic records and imaging results documenting the treatment course, including baseline enhanced chest computed tomography (CT), abdominal CT, brain magnetic resonance imaging, bone scanning, and positron emission tomography‐CT (PET‐CT), as well as chest CT scans following every two cycles of induction immunochemotherapy and post‐treatment response evaluation CT scans for patients undergoing CRT. Patients were excluded if they had: (1) concurrent other malignancies; (2) active or previous autoimmune diseases; (3) undergone lobectomy or pneumonectomy, anti‐cytotoxic T lymphocyte‐associated protein 4 therapy, targeted therapy, or palliative treatment; (4) identified *EGFR* mutations, or *ALK* and *ROS1* rearrangements at baseline. Notably, patients who were initially assessed as resectable but ultimately missed the opportunity for radical surgery were also included in the study. In the process of enrolling patients, clinical information and treatment outcomes for those who only received CRT were also collected. This was done to compare the efficacy and toxic reactions with patients who underwent induction immunochemotherapy. However, the patients who underwent only CRT were not the target population of this study. The research received approval from the Institutional Review Boards at each of the three involved hospitals and adhered to the principles of the Declaration of Helsinki, as updated in 2013. All participants provided written consent for their clinical data to be utilized in this study.

### 5.2 Treatment procedures

The specific duration of the induction ICI combined with chemotherapy was determined by the attending clinicians, and no additional selection was made for patients who received more than two cycles. Patients without distant metastasis during the induction treatment were treated with definitive concurrent or sequential CRT with curative intent, which involved a prescribed dose of over 54 Gy of conventional fractionated intensity‐modulated radiotherapy, and at least two cycles of platinum‐based chemotherapy.^5^ Those who did not develop distant metastasis during CRT were eligible to either proceed with consolidation ICI or opt‐out. Clinicians tailor the treatment strategy to the patient's specific conditions. For the final analysis, all stage III unresectable NSCLC patients who underwent at least two cycles of induction ICI with chemotherapy were included.

### 5.3 Follow‐up and data collection

Electronic medical records were carefully reviewed for all included patients. Baseline data collected included gender, age, ECOG performance status, smoking history, histology, and tumor staging. Follow‐up evaluations after the treatment were scheduled every three months for the first 2 years, and every 6 months for the subsequent 3 years. The information on treatment efficacy and toxicity was elaborately collected from follow‐up and imaging reports for the final analyses. Survival data for patients lost to follow‐up were regarded as censored data. The last follow‐up was on October 15, 2023.

### 5.4 Outcomes

The study's primary endpoint was to evaluate the survival benefits of the treatment approach, focusing on OS and PFS. OS and PFS were calculated from the beginning of induction immunochemotherapy, with PFS defined as the duration from induction immunochemotherapy initiation to the first occurrence of local‐regional progression, distant metastasis, or death, inclusive of disease progression during therapy and post‐treatment recurrence. OS was defined as the time from the start of treatment until death. The secondary endpoints included the ORR and DCR of induction ICI combined with chemotherapy, ORR of CRT, and the assessment of toxicities. Treatment responses were assessed from the baseline based on the Response Evaluation Criteria in Solid Tumors version 1.1, and toxicities were classified and graded according to the National Cancer Institute Common Terminology Criteria for Adverse Events version 5.0.

### 5.5 Statistical analysis

Demographic and clinical features at baseline were summarized with median values and IQR for continuous variables, and by using proportions for categorical variables. PFS and OS estimates were derived using the Kaplan‐Meier method, with median values and their 95% CIs calculated, as well as survival probabilities. The Cox proportional hazards regression models were utilized to investigate the relationship between various relevant variables and survival outcomes. Patients with missing baseline characteristics and treatment data were eliminated in regression analyses. Multivariate analysis included variables that had a *p*‐value < 0.1 in the univariate analysis. HRs and their 95% CIs were determined, with a two‐sided *p*‐value < 0.05 considered statistically significant. Data analysis was conducted using SPSS version 23.0 (IBM Corp.) and R programming software (version 4.2.3).

## AUTHOR CONTRIBUTIONS

L.L.W., B.C., and Z.S.Z. drafted the manuscript and prepared the figures and tables. X.J.S., J.J.K., and S.X.R. revised the manuscript. Y.C., Q.H.X., S.Y.Y., and Y.J.Y. collected the related references. L.L.W., C.C.Z., and Y.P.X. designed the study. All authors read and approved the final manuscript. L.L.W., B.C., and X.J.S. contributed equally to this work. All authors have read and approved the final manuscript.

## CONFLICT OF INTEREST STATEMENT

The authors declare no conflict of interest. The figures were created with the help of BioRender.com.

## ETHICS STATEMENT

The authors are accountable for all aspects of the work in ensuring that questions related to the accuracy or integrity of any part of the work are appropriately investigated and resolved. The study was conducted in accordance with the Declaration of Helsinki (as revised in 2013). The study was approved by the Ethics Committee of Shanghai Pulmonary Hospital (19231ZL), Zhejiang Cancer Hospital (IRB‐2021‐179), and Shandong University Qilu Hospital (KYLL‐2023(ZM)−450). Written informed consent was taken from all the patients.

## Supporting information

Supporting Information

Supporting Information

## Data Availability

All data used and/or analyzed in the study are available in the supplementary materials or from the corresponding author upon reasonable request.
