# Supplementary material for: Induction immunochemotherapy followed by definitive chemoradiotherapy for unresectable locally advanced non‐small cell lung cancer: a multi‐institutional retrospective cohort study
Source: MedComm (2020). 2024 Mar 2;5(3):e501. doi: 10.1002/mco2.501 (PMC10908364; doi:10.1002/mco2.501)
Supplement: Supplementary file 1 — Supporting Information [file MCO2-5-e501-s001.docx]

**Induction immunochemotherapy followed by definitive chemoradiotherapy for unresectable locally advanced non-small cell lung cancer: A multi-institutional retrospective cohort study**

Leilei Wu^1,2#^, Bo Cheng^3#^, Xiaojiang Sun^2#^, Zhenshan Zhang^4^, Jingjing Kang^1^, Yun Chen^1^, Qinghua Xu^1^, Shuangyan Yang^1^, Yujie Yan^1^, Shengxiang Ren^5^, Caicun Zhou^5*^, Yaping Xu^1*^

1. Department of Radiation Oncology, Shanghai Pulmonary Hospital, School of Medicine, Tongji University, Shanghai, China.
2. Department of Radiation Oncology, Cancer Hospital of University of Chinese Academy of Sciences (Zhejiang Cancer Hospital), Institute of Cancer and Basic Medicine (IBMC), Chinese Academy of Sciences, Hangzhou, China.
3. Department of Radiation Oncology, Qilu Hospital, Cheeloo College of Medicine, Shandong University, Jinan, Shandong, China.
4. Department of Radiation Oncology, Shanghai Proton and Heavy Ion Center, Fudan University Cancer Hospital, Shanghai, China.
5. Department of Medical Oncology, Shanghai Pulmonary Hospital, School of Medicine, Tongji University, Shanghai, China.

# These authors contributed equally to this work and share first authorship.

*Correspondence: Yaping Xu, Caicun Zhou.

Yaping Xu, E-mail: [xuyaping1207@126.com](mailto:xuyaping1207@126.com)

Caicun Zhou, E-mail: [caicunzhoudr@163.com](mailto:caicunzhoudr@163.com)

**Key words:**

Induction immunochemotherapy, definitive chemoradiotherapy, unresectable LA-NSCLC, survival


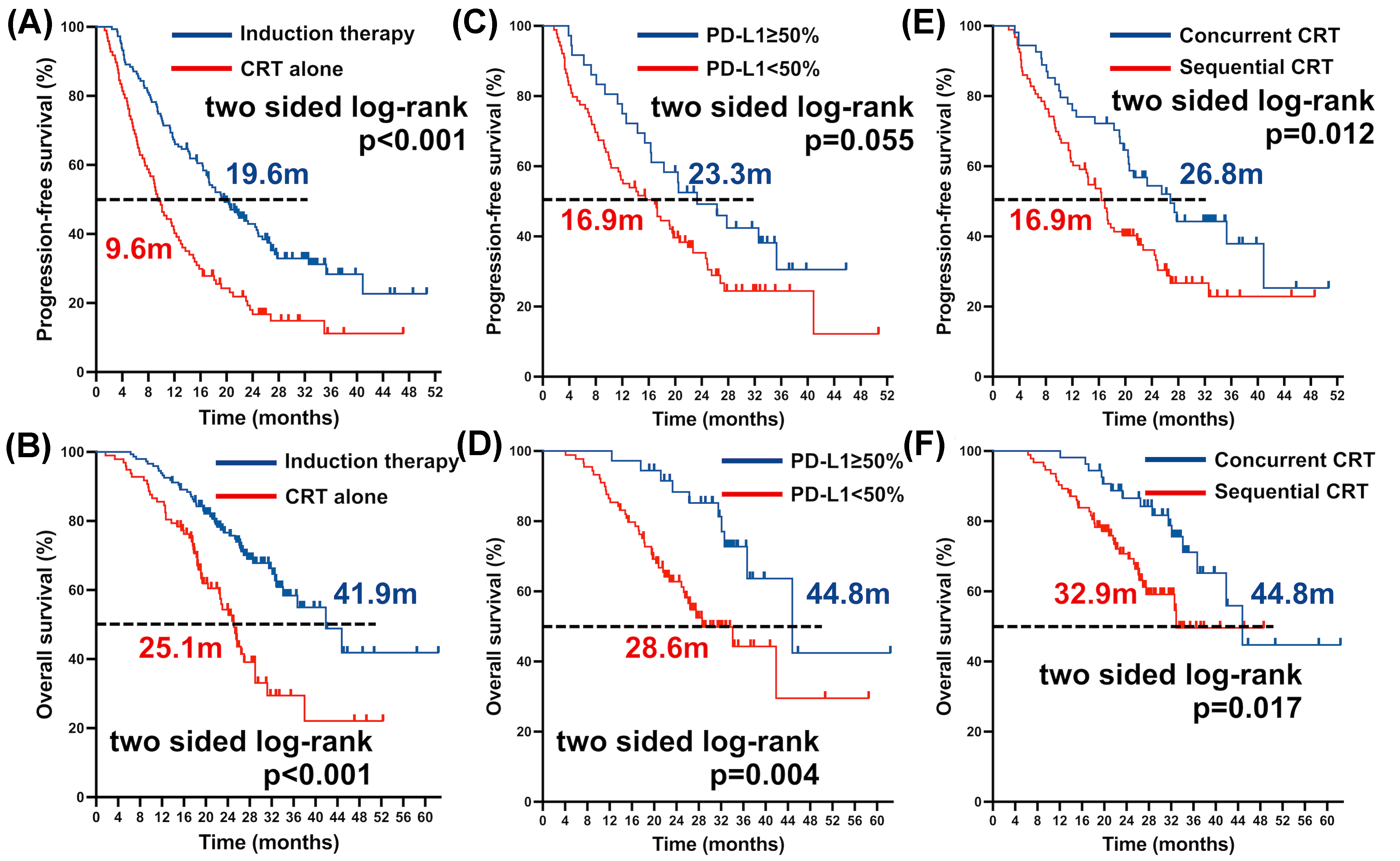


**Supplementary Figure 1** Comparison of survival curves among subgroups. (A-B) Comparison of PFS and OS between patients receiving induction immunochemotherapy followed by CRT (n=147) and patients who received only CRT. (C-D) Comparison of PFS and OS in all 158 patients with induction immunochemotherapy using a PD-L1 cut-off value of 50%. (E-F) Comparison of PFS and OS between concurrent and sequential CRT in all 147 patients receiving induction immunochemotherapy followed by CRT. Abbreviations: CRT, chemoradiotherapy; PD-L1, programmed death ligand-1

**Supplementary Table 1** Comparison of best responses to induction immunochemotherapy using PD-L1 cut-off values of 1% and 50%

| **Response rates** | **PD-L1 expression** | | | | **p value** | |
| --- | --- | --- | --- | --- | --- | --- |
|  | <1% (n=47) | 1-49% (n=42) | ≥50% (n=36) | Missing (n=33) | <1% vs. ≥1% | <50% vs. ≥50% |
| CR | 0 (0.0) | 0 (0.0) | 1 (2.8) | 0 (0.0) | 1.000 | 0.288 |
| PR | 22 (46.8) | 20 (47.6) | 25 (69.4) | 15 (45.5) | 0.237 | **0.024*** |
| SD | 17 (36.2) | 13 (31.0) | 7 (19.4) | 12 (36.4) | 0.212 | 0.114 |
| PD | 8 (17.0) | 9 (21.4) | 3 (8.3) | 6 (18.2) | 0.809 | 0.137 |
| Objective response | 22 (46.8) | 20 (47.6) | 26 (72.2) | 15 (45.5) | 0.186 | **0.011*** |
| Disease control | 39 (83.0) | 33 (78.6) | 33 (91.7) | 27 (81.8) | 0.809 | 0.137 |

Abbreviations: CR, complete response; PR, partial response; SD, stable disease; PD, progressive disease; LRP, local-reginal progression; DM, distant metastasis; CRT, chemoradiotherapy; PD-L1, programmed death ligand-1. *p<0.05.

**Supplementary Table 2** Univariate and multivariate analysis of survival outcomes

| **Variables** | **PFS** | | | | **OS** | | | |
| --- | --- | --- | --- | --- | --- | --- | --- | --- |
|  | Univariate analysis | | Multivariate analysis | | Univariate analysis | | Multivariate analysis | |
|  | HR (95% CI)† | p value | HR (95% CI) | p value | HR (95% CI) | p value | HR (95% CI) | p value |
| Age |  |  |  |  |  |  |  |  |
| <65 vs. ≥65 | 0.786 (0.535-1.155) | 0.221 |  |  | 0.827 (0.494-1.386) | 0.472 |  |  |
| Sex |  |  |  |  |  |  |  |  |
| Male vs. Female | 0.969 (0.488-1.923) | 0.929 |  |  | 0.839 (0.361-1.952) | 0.684 |  |  |
| Smoking history |  |  |  |  |  |  |  |  |
| Never vs. Former or current | 0.672 (0.420-1.075) | 0.097 | 0.461 (0.254-0.836) | 0.011* | 0.718 (0.373-1.383) | 0.322 |  |  |
| ECOG performance status |  |  |  |  |  |  |  |  |
| 0 vs. 1-2 | 0.816 (0.543-1.228) | 0.330 |  |  | 0.675 (0.387-1.177) | 0.166 |  |  |
| Tumor histological type |  |  |  |  |  |  |  |  |
| Squamous vs. Non-squamous | 0.744 (0.494-1.121) | 0.158 |  |  | 1.207 (0.686-2.122) | 0.514 |  |  |
| TNM stage |  |  |  |  |  |  |  |  |
| IIIA vs. IIIB and IIIC | 0.686 (0.447-1.053) | 0.085 | 0.869 (0.519-1.454) | 0.593 | 0.824 (0.474-1.433) | 0.493 |  |  |
| Tumoral PD-L1 status |  |  |  |  |  |  |  |  |
| <1% vs. ≥1% | 1.193 (0.767-1.856) | 0.434 |  |  | 1.566 (0.889-2.761) | 0.121 |  |  |
| <50% vs. ≥50% | 1.609 (0.986-2.625) | 0.057 | 1.386 (0.820-2.343) | 0.223 | 2.673 (1.325-5.394) | 0.006** | 2.534 (0.900-7.133) | 0.078 |
| Cycles of induction therapy |  |  |  |  |  |  |  |  |
| 2-3 vs. ≥4 | 0.720 (0.490-1.057) | 0.093 | 0.479 (0.295-0.776) | 0.003** | 0.810 (0.487-1.348) | 0.417 |  |  |
| Sequence of CRT |  |  |  |  |  |  |  |  |
| Sequential vs. Concurrent | 1.737 (1.123-2.687) | 0.013* | 2.075 (1.247-3.456) | 0.005** | 2.090 (1.125-3.882) | 0.020* | 2.944 (1.149-7.547) | 0.025* |
| Radiotherapy dose |  |  |  |  |  |  |  |  |
| <60 vs. ≥60 Gy | 1.519 (1.008-2.289) | 0.045* | 1.420 (0.889-2.268) | 0.142 | 1.269 (0.723-2.227) | 0.406 |  |  |
| Duration of consolidation ICI |  |  |  |  |  |  |  |  |
| ≤12 vs. >12 months | 1.370 (0.830-2.259) | 0.218 |  |  | 2.315 (1.022-5.242) | 0.044* | 2.311 (0.951-5.615) | 0.065 |

Abbreviations: PFS, progression-free survival; OS, overall survival; HR, hazard ratio; CI, confidence interval; ECOG, Eastern Cooperative Oncology Group; PD-L1, programmed death ligand-1; ICI, immune checkpoint inhibitors. †The reference category is the latter category. *p<0.05, **p<0.01.
