# Supplementary figures and images for: Induction immunochemotherapy followed by definitive chemoradiotherapy for unresectable locally advanced non‐small cell lung cancer: a multi‐institutional retrospective cohort study
Source: MedComm (2020). 2024 Mar 2;5(3):e501. doi: 10.1002/mco2.501 (PMC10908364; doi:10.1002/mco2.501)

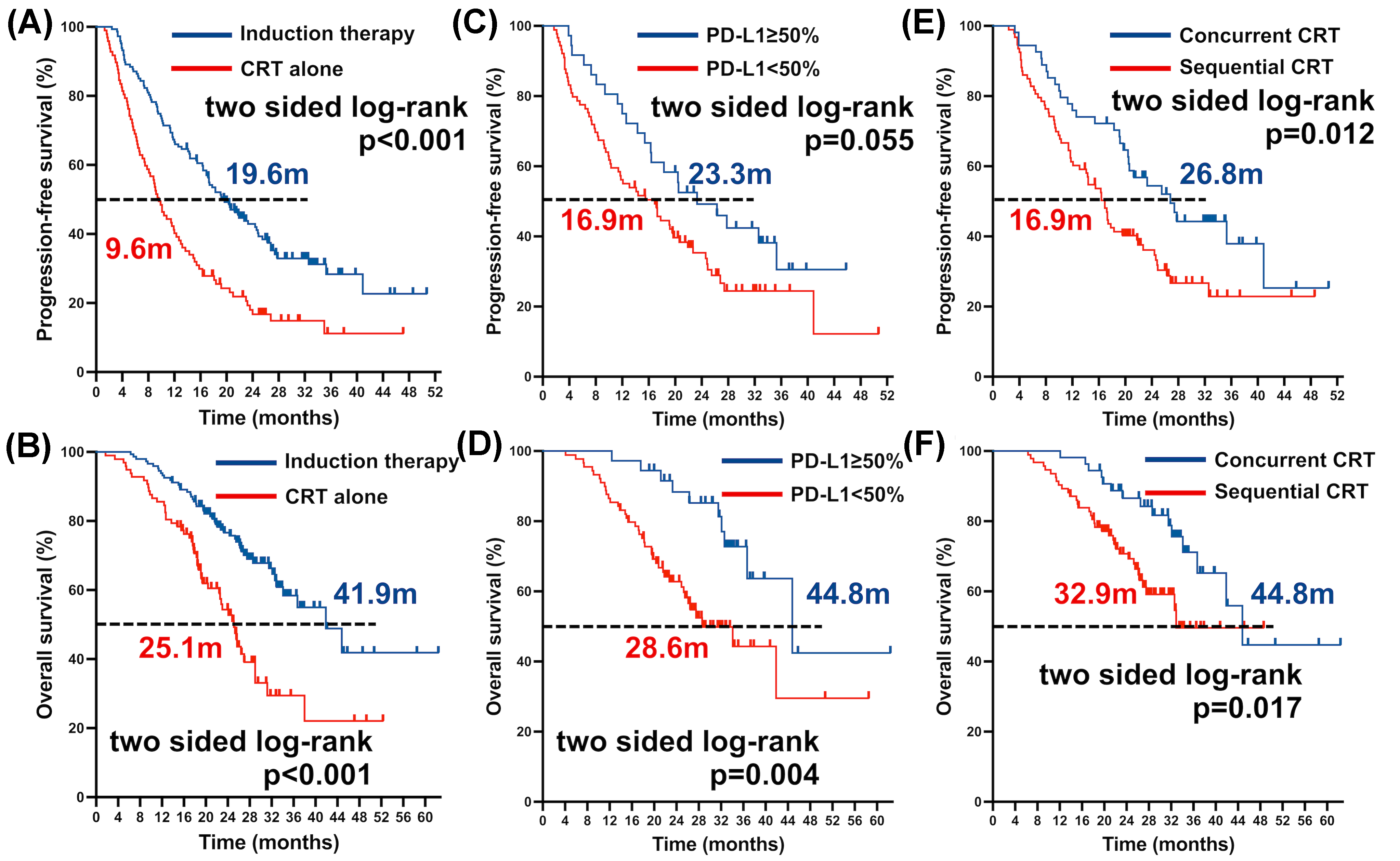

Supplement: Supplementary file 2 — Supporting Information [file MCO2-5-e501-s002.png]
